# A Body Shape Index and Body Roundness Index in Relation to Anxiety, Depression, and Psychological Distress in Adults

**DOI:** 10.3389/fnut.2022.843155

**Published:** 2022-04-25

**Authors:** Keyhan Lotfi, Ammar Hassanzadeh Keshteli, Parvane Saneei, Hamid Afshar, Ahmad Esmaillzadeh, Peyman Adibi

**Affiliations:** ^1^Department of Community Nutrition, School of Nutritional Sciences and Dietetics, Tehran University of Medical Sciences, Tehran, Iran; ^2^Department of Medicine, University of Alberta, Edmonton, AB, Canada; ^3^Department of Community Nutrition, Food Security Research Center, School of Nutrition and Food Science, Isfahan University of Medical Sciences, Isfahan, Iran; ^4^Psychosomatic Research Center, Isfahan University of Medical Sciences, Isfahan, Iran; ^5^Obesity and Eating Habits Research Center, Endocrinology and Metabolism Molecular-Cellular Sciences Institute, Tehran University of Medical Sciences, Tehran, Iran; ^6^Department of Community Nutrition, School of Nutrition and Food Science, Isfahan University of Medical Sciences, Isfahan, Iran; ^7^Isfahan Gastroenterology and Hepatology Research Center, Isfahan University of Medical Sciences, Isfahan, Iran

**Keywords:** A Body Shape Index, Body Roundness Index, psychological distress, depression, anxiety, psychological disorders

## Abstract

**Background:**

Despite the large evidence on the association between obesity and psychological disorders, studies investigating new anthropometric indices in relation to mental health are limited. We aimed to explore the association between A Body Shape Index (ABSI) and Body Roundness Index (BRI) and common psychological disorders (anxiety, depression, and psychological distress) among Iranian adults.

**Methods:**

In this cross-sectional investigation, anthropometric measures of 3213 Iranian adults were gathered using a validated self-reported questionnaire. ABSI and BRI values of participants were calculated through pre-defined formulas. General Health Questionnaire (GHQ) and Hospital Anxiety and Depression Scale (HADS) validated for Iranians were used to assess psychological distress, anxiety, and depression.

**Results:**

Mean age of participants was 36.6 ± 7.73, and 62.8% of them were women. ABSI and BRI were higher in subjects with anxiety and psychological distress. Also, depressed participants had higher BRI. After considering potential confounders, individuals in the last tertile of ABSI, compared to the first tertile, had higher odds of anxiety (OR: 1.41, 95%CI: 1.04, 1.93) and psychological distress (OR: 1.39, 95%CI: 1.09, 1.79). Also, a marginal association was found between the highest category of ABSI and depression (OR: 1.27; 95%CI: 1.00, 1.61). In the sex-stratified analysis, ABSI was positively related to odds of anxiety (OR: 1.58; 95%CI; 1.12, 2.22), depression (OR: 1.40; 95%CI; 1.07, 1.84), and psychological distress (OR: 1.51; 95%CI; 1.13, 2.01) among women, but not men. We failed to find any significant association between BRI and depression, anxiety and psychological distress.

**Conclusion:**

We found that ABSI was associated with anxiety, depression and psychological distress among females, but not males. However, we did not find a significant relation between BRI and the outcomes. Further prospective studies are required to confirm our findings.

## Introduction

Mental health is known as a state of well-being that a person apprehends his or her capacity, can handle life difficulties and can involve in the community (1). The prevalence of mental disorders is increasing drastically, of which depression and anxiety are the most considerable (2, 3). Approximately, 4.4 and 4.8% of the world's population is, respectively, affected by depression and anxiety (4, 5). According to a national survey, the prevalence of depression and anxiety in Iranian adults was 13.6–42.6 and 15.6%, respectively (6, 7).

Some previous investigations have focused on the association between body mass index (BMI) and the risk of mental disorders (8–10). BMI was inversely associated with depressive disorders in some populations (10); however, other reports showed no significant association (9). Furthermore, previous meta-analyses found a positive association between BMI and depression and anxiety (11, 12). There are several limitations for BMI, as an index of body composition, such as its inability to distinguish between muscle mass and fat mass as well as the inability to determine fat locations (13). Differential relations between the distribution of body fat (general vs. abdominal obesity, or visceral vs. subcutaneous fat) and anxiety and depression were previously suggested (14). Hypothalamic-pituitary-adrenocortical (HPA) axis over-activity is a possible biological mechanism by which depression and anxiety could be developed, and this over-activity could particularly result from abdominal obesity (15). Furthermore, among individuals with abdominal obesity, metabolic complications are more prevalent in those with greater visceral fat than in those with higher subcutaneous fat (16). Therefore, not only the type of obesity, but also the distribution of body fat is an important factor for developing anxiety and depression.

Due to the important limitations of BMI, the association between some other indices, such as total body fat, triceps skin-folds, waist circumference (WC) and waist-hip ratio (WHR), and the risk of depression have been investigated (9, 10). Although total body fat and triceps skin-folds were positively linked to depression (9), WHR and WC were not associated with these conditions in some societies (9, 10, 17). A meta-analysis revealed a 38% increased odds of depression in males and females with enlarged WC (18). Nevertheless, WC has also key limitations of being sensitive to body size, fat percentage and fat distribution as well as being correlated with BMI (13). A Body Shape Index (ABSI) has been developed to compensate for BMI and WC limitations and discrepancies (13). This index was proposed to predict mortality risk, independent of BMI (13). Higher ABSI contributes to a more fraction of visceral fat, indicating that WC is higher than expected for a given height and weight (13). Additionally, Body Roundness Index (BRI) is a novel anthropometric index developed to predict both percentages of total body fat and visceral fat, which are not clearly addressed by BMI (19). The association between BRI and odds of CVD and CVD risk factors has been previously shown (20). Both ABSI and BRI were established to detect abdominal obesity, and predominantly visceral fat. To find the more reliable predictor, previous studies have investigated both ABSI and BRI in relation to the outcomes and found inconsistent results (21–24).

Adiposity types differently affect anxiety and depression status (14), and traditional indices have some limitations for differentiating general and abdominal obesity as well as visceral and subcutaneous fat. ABSI and BRI have high correlation with visceral fat (13), which is found to be more important in the prevalence of mental disorders. There is a lack of evidence on the association between ABSI and BRI and mental health. Therefore, this study aimed to examine the relationship between ABSI and BRI and the prevalence of common psychological disorders (anxiety, depression, and psychological distress) in a large population of Iranian adults.

## Materials and Methods

### Participants

The current cross-sectional investigation was carried out on data from the Study on the Epidemiology of Psychological, Alimentary Health and Nutrition (SEPAHAN) project. The methodology of the SEPAHAN project has already been described elsewhere (25). The original project recruited a large number of Iranian men and women (aged ≥18 y) occupied in 50 different health centers in Isfahan, Iran. The target population was both health providers and those involved in administrative tasks. Data were collected in two separate stages (3–4 weeks apart) with two different sets of questionnaires. In the first stage (April 2010), demographic characteristics, medical history, and anthropometric measures of the subjects were collected by a self-reported questionnaire. In this phase, questionnaires were distributed and collected within three weeks. The second stage (mid-May 2010) was designed to assess psychological health of the participants, and was completed in two weeks. Participants were also provided the study's brochures to help them filling out the questionnaires. Written informed consent forms were completed by all participants before data collection. The Medical Research Ethics Committee of Isfahan University of Medical Sciences, Isfahan, Iran, was ethically approved the SEPAHAN project.

### Anthropometric Assessment

Participants' weight, height, and WC were obtained by using a self-reported questionnaire (26). BMI was then computed by dividing weight (kg) and height squared (m^2^). A pilot study on 171 individuals examined the validity of the self-reported anthropometric values (26). This validation study compared self-reported values for weight, height, and WC with the values measured by a trained nutritionist. The correlation coefficients for the self-reported height, weight and WC versus measured values were 0.83 (*P* < 0.001), 0.95 (*P* < 0.001), and 0.60 (*P* < 0.001), respectively. Furthermore, the correlation between BMI values calculated from self-reported and nutritionist-measured values was 0.70 (*P* < 0.001). These findings indicate a reasonable measure for anthropometric indices provided by the self-reported questionnaire.

ABSI and BRI were calculated by previously mentioned formulas (13, 20) using WC (m), BMI (kg/m^2^), and height (m) as below:


$$\begin{array}{lll} \text{ABSI} & = & \frac{\text{WC}}{\text{BM}{\text{I}}^{2/3}\text{heigh}{\text{t}}^{1/2}}\\ \text{BRI} & = & 364.2-365.5\;\times \sqrt{1-\left(\frac{{\left(\frac{\text{WC}}{2\text{π}}\right)}^{2}}{{\left(0.5\text{ height}\right)}^{2}}\right)} \end{array}$$

ABSI and BRI were not previously validated against gold-standard measurements such as Magnetic resonance imaging (MRI), Computed tomography (CT) or Dual-energy X-ray absorptiometry (DEXA), because these measurement methods are expensive, unpractical and hard to use in large populations.

### Assessment of Common Psychological Disorders

Anxiety and depression were examined using the Iranian validated version of Hospital Anxiety and Depression Scale (HADS) (27). This questionnaire contains 14 items by which anxiety and depression are separately examined as an individual subscale. Subjects were received a score of 0 to 21. For each subscale, values of 0 to 7 were defined as having normal mental health, and scores ≥8 were considered as psychological disorders (28). A study conducted on 167 Iranian adults indicated the reasonable validity of the translated version of HADS (27).

We used the Iranian validated version of General Health Questionnaire (GHQ) to screen psychological distress (29). This questionnaire contains 12 items, and each item contains a 4-point rating scale (less than usual, no more than usual, rather more than usual, or much more than usual). The bimodal scoring method (0-0-1-1) was applied to define psychological distress. By using this method, a score between 0-12 belongs to each participant; higher values define a higher degree of psychological distress (30). In the current investigation, scores ≥4 were considered as high psychological distress. GHQ-12 has a reasonable validity in Iranian adults, according to a preliminary study (29).

### Assessment of Other Variables

We applied a self-administered questionnaire to gather data on sex, age, marital status (single/married/divorced or widowed), smoking status (non-smoker/former smoker/current smoker), diabetes (yes/no), and use of anti-psychotic medications (including sertraline, nortriptyline, imipramine or amitriptyline, fluvoxamine, fluoxetine, and citalopram). Physical activity level was assessed by the General Practice Physical Activity Questionnaire (GPPAQ). This questionnaire classifies individuals into four levels of activity (31); inactive (no physical activity), moderately inactive (<1 h/week), moderately active (1–3 h/week), and active (≥3 h/week).

### Statistical Analysis

After computing ABSI and BRI, tertile cut-off points of these indices were used to classify study participants. Continuous and categorical variables for general characteristics of subjects were reported as means ± SDs and percentages, respectively. Analysis of variance (ANOVA) and Chi-square tests were applied to determine differences across ABSI and BRI tertile. Correlation coefficients of ABSI with BRI, WC, height, weight, and BMI were obtained while age and sex were adjusted. Independent sample t-test was used to examine differences in ABSI and BRI among individuals with and without anxiety, depression and/or psychological distress. We evaluated the association of ABSI and BRI with common psychological disorders by logistic regression in crude and multivariable adjusted models. Sex (male/female), age (continuous), marital status (single/married/divorced or widowed), smoking status (non-smoker/former smoker/current smoker), physical activity (inactive/moderately inactive/moderately active/active), diabetes (yes/no), and use of anti-psychotic medications (yes/no) were adjusted. The first tertile of ABSI/BRI was considered as the reference for all odds ratios. Furthermore, by considering the tertiles of ABSI/BRI as an ordinal variable, the trend of odds ratios across these tertiles was estimated. Sensitivity analysis was also performed by excluding anti-psychotic medication users. Furthermore, stratified analysis based on sex (male/female) was also performed. We also performed a linear regression analysis by considering ABSI, BRI and scores of psychological disorders as continuous variables. As the ABSI values had a narrow range (0.0341 to 0.1180) and were small, we multiplied the original values by 100 to have more intuitive results. Standardized regression coefficients were also reported. Variance Inflation Factor (VIF) was measured to assess the multi-collinearity of the predictors in the adjusted model. SPSS software (version 20; SPSS Inc., Chicago IL) was applied for all statistical analyses, and *P* < 0.05 was considered as significant level.

## Results

Questionnaires were sent to 10087 individuals in both phases, with a response rate of 86.16% (*n* = 8,691) in the first and 61.85% (*n* = 6,239) in the second phase. Then, data of two stages were combined. Subjects were excluded if they did not have information at phase 1 or 2, did not report their identification code in phase 1 or 2, or did not have data on exposure, outcome, or covariates. Finally, 3,213 individuals had the complete data for inclusion in the current analysis. Participants had a mean age and weight of 36.6 ± 7.73 (y) and 68.37 ± 13.09 (kg), respectively. Females made up 62.8% of the study participants. General characteristics of study participants based on ABSI and BRI tertiles are indicated in Table 1. Compared to the first tertile, subjects in the last tertile of ABSI had higher age, weight, as well as lower BMI. Also, individuals in the top tertile of ABSI were more likely to be married and have diabetes; and less likely to be female, smoker, and physically active compared to the bottom tertile. Furthermore, participants in the third tertile of BRI had higher age, weight and BMI, and were more likely to be females, married, diabetic, anti-psychotic drugs user, and overweight/obese compared to the first tertile. In contrast, adults in the last tertile of BRI, compared to the first one, had lower physical activity levels.

**Table 1:** General characteristics of study participants across tertiles of ABSI and BRI (*n* = 3,213)^a^.

	**Tertiles of ABSI**				**Tertiles of BRI**			
	**T**_**1**_ **(*****n** **=*** **1,071)**	**T**_**2**_ **(*****n** **=*** **1,071)**	**T**_**3**_ **(*****n** **=*** **1,071)**	***P* ^b^**	**T**_**1**_ **(*****n** **=*** **1,071)**	**T**_**2**_ **(*****n** **=*** **1,072)**	**T**_**3**_ **(*****n** **=*** **1,070)**	***P* ^b^**
Age (y)	35.2 ± 7.44	36.6 ± 7.49	38.0 ± 8.01	<0.001	34.1 ± 7.53	36.6 ± 7.49	39.1 ± 7.36	<0.001
Weight (kg)	67.1 ± 13.69	69.1 ± 12.50	68.9 ± 12.97	0.001	65.1 ± 13.32	68.7 ± 13.06	71.2 ± 12.13	<0.001
Body mass index (kg/m^2^)	25.4 ± 5.54	25.2 ± 3.87	24.8 ± 3.78	0.02	22.4 ± 3.31	24.8 ± 3.01	28.1 ± 4.88	<0.001
Female (%)	74.9	58.2	55.3	<0.001	52.2	59.9	76.3	<0.001
Married (%)	75.6	84.7	87.0	<0.001	72.2	85.6	89.6	<0.001
Diabetes (%)	0.6	1.7	2.4	0.002	0.7	1.2	2.8	<0.001
Anti-psychotic medications^c^ (%)	4.2	4.9	6.3	0.09	3.5	4.9	7.1	0.001
Smokers (%)	14.5	13.1	13.3	0.01	13.2	12.4	15.2	0.31
Physically active (%) (active)	31.4	26.0	26.7	0.01	34.3	29.7	19.7	<0.001
Overweight/Obese^d^ (%)	47.2	48.6	48.0	0.81	18.0	46.9	79.0	<0.001

a *All values are means ± standard deviation (SD), unless indicated*.

b *Obtained from ANOVA for continuous variables and chi-square test for categorical variables*.

c *Anti-psychotic medications included the intake of nortriptyline, amitriptyline or imipramine, fluoxetine, citalopram, fluvoxamine and sertraline*.

d *BMI ≥ 25 kg/m^2^*.

**Figure 1:**
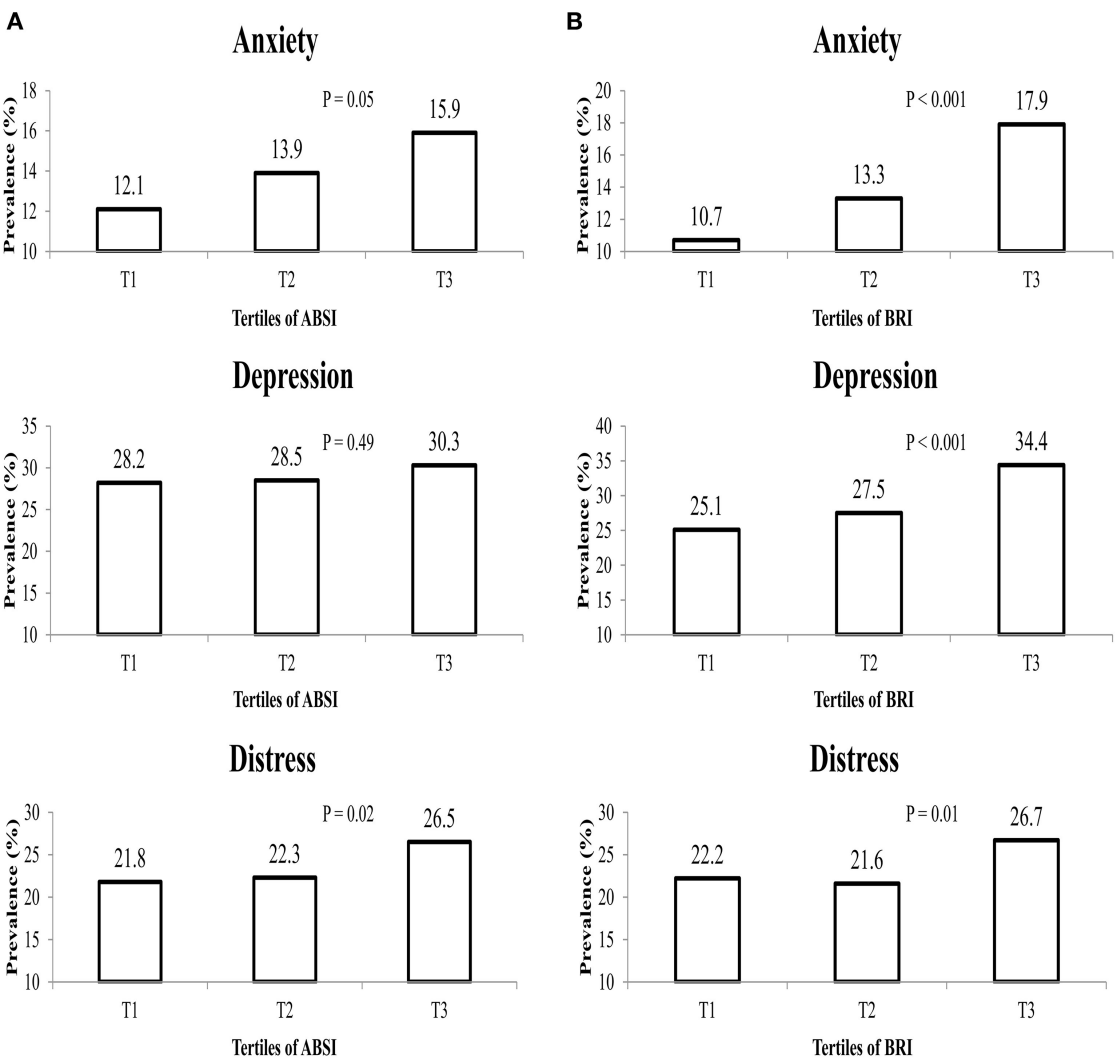
The prevalence of anxiety, depression and distress in study participants across tertiles of **(A)** ABSI, and **(B)** BRI.

The overall prevalence of anxiety, depression, and psychological distress was 14, 29, and 23.5%, respectively. Figure 1 illustrates the prevalence of anxiety, depression, and psychological distress across tertiles of ABSI and BRI. Individuals in the top category of ABSI, in comparison to the reference category, had a higher prevalence of anxiety (15.9% vs. 12.1%; *P* = 0.05) and distress (25.6% vs. 21.8%; *P* = 0.02). However, there were no significant differences in the prevalence of depression across tertiles of ABSI (*P* = 0.49). Furthermore, subjects in the last tertile of BRI had a higher prevalence of anxiety (17.9% vs. 10.7%; *P* < 0.001), depression (34.4% vs. 25.1%; *P* < 0.001), and distress (26.7% vs. 22.2%; *P* = 0.01) compared to the first tertile.

Pearson correlation coefficients between anthropometric indices of study participants are reported in Table 2. ABSI had a modest correlation with WC (r = 0.58) and BRI (r = 0.35). However, weak correlations were found between ABSI and height, weight, or BMI (|r| ≤ 0.20). Furthermore, BRI showed a relatively high correlation with WC (r = 0.72) and BMI (r = 0.74); besides a modest correlation with height (|r| = 0.57) and weight (r = 0.32).

**Table 2:** Pearson correlation coefficients between anthropometric indices of study participants^a^.

	**Height**	**Weight**	**WC**	**BMI**	**ABSI**	**BRI**
Height	1					
Weight	0.36	1				
WC	0.08	0.67	1			
BMI	−0.26	0.77	0.61	1		
ABSI	0.09	−0.12	0.58	−0.20	1	
BRI	−0.57	0.32	0.72	0.74	0.35	1

*WC, Waist Circumference; BMI, Body Mass Index; ABSI, A Body Shape Index; BRI, Body Roundness Index*.

a *Correlation coefficients between height, weight, BMI, WC, ABSI and BRI among 3,213 adults, adjusted for age and sex (all P-values were < 0.001)*.

Table 3 indicates the differences of ABSI and BRI values among individuals with and without anxiety, depression, and psychological distress. Individuals suffering from anxiety and distress had significantly higher ABSI values than healthy subjects. However, no significant difference was observed between depressed and non-depressed participants (*P* = 0.20). Furthermore, higher mean value of BRI was observed among individuals with anxiety (*P* < 0.001), depression (*P* < 0.001), and distress (*P* = 0.003).

**Table 3:** ABSI and BRI values of the study participants, based on their psychological health status^a^.

	**Anxiety**			**Depression**			**Distress**		
	**Yes**	**No**	***P***	**Yes**	**No**	***P***	**Yes**	**No**	***P***
ABSI (m^11/6^kg^−2/3^)	0.0806 ± 0.0071	0.0796 ± 0.0069	0.006	0.0800 ± 0.0071	0.0797 ± 0.0069	0.20	0.0803 ± 0.0071	0.0796 ± 0.0069	0.01
BRI	4.97 ± 1.19	4.72 ± 1.01	<0.001	4.88 ± 1.10	4.70 ± 1.02	<0.001	4.85 ± 1.11	4.72 ± 1.02	0.003

a *All values are means ± standard deviation (SD) obtained from independent Samples T-test*.

Crude and multivariable-adjusted odds ratios for anxiety, depression, and psychological distress across tertiles of ABSI and BRI are presented in Table 4. In the crude model, participants in the last tertile of ABSI, compared to the first one, were more likely to have anxiety (OR: 1.37; 95%CI: 1.07, 1.75) and high psychological distress (OR: 1.30; 95%CI: 1.06, 1.59). However, no significant association was observed between ABSI and odds of depression (OR: 1.11; 95%CI: 0.92, 1.34) in the crude model. After considering age, sex, marital status, diabetes, smoking, physical activity and anti-psychotic medications as the potential confounders, ABSI was significantly associated with odds of anxiety (OR for T3 vs. T1: 1.41; 95%CI: 1.04, 1.93) and psychological distress (OR for T3 vs. T1: 1.39; 95%CI: 1.09, 1.79). Furthermore, a marginal significant relation was observed between ABSI and the possibility of being depressed in the multivariable-adjusted model (OR for T3 vs. T1: 1.27; 95%CI: 1.00, 1.61). In the crude model, participants in the top tertile of BRI, compared to the bottom, had 81%, 56%, and 28% higher odds of anxiety (95%CI: 1.41, 2.32), depression (95%CI: 1.30, 1.88), and high psychological distress (95%CI: 1.04, 1.56), respectively. However, after considering potential confounders, results were attenuated and no significant relation was found. A significant trend was observed for all mental disorders across tertiles of ABSI, but not for BRI, in the adjusted model. In all observed associations, sex, physical activity and use of anti-psychotic medications had the most confounding effects. In addition, smoking status had a high confounding effect for the association between ABSI, BRI and depression. After excluding individuals using anti-psychotic medications (*n* = 84), a significant association between ABSI and odds of depression was observed (OR: 1.30; 95%CI: 1.02, 1.67), and other findings did not alter (Supplementary Table 1).

**Table 4:** Multivariable- adjusted odds ratio for anxiety, depression, and distress across tertiles of ABSI and BRI (*n* = 3,213)^a^.

	**Tertiles of ABSI**				**Tertiles of BRI**			
	**T_**1**_**	**T_**2**_**	**T_**3**_**	**P_**trend**_**	**T_**1**_**	**T_**2**_**	**T_**3**_**	**P_**trend**_**
**Anxiety**								
Participants/Cases (*n*)	1,071/130	1,071/149	1,071/170		1,071/115	1,072/143	1,070/191	
Crude	1.00	1.17 (0.91–1.51)	1.37 (1.07–1.75)	0.01	1.00	1.28 (0.99–1.66)	1.81 (1.41–2.32)	<0.001
Adjusted^†^	1.00	1.30 (0.95–1.76)	1.41 (1.04–1.93)	0.03	1.00	1.06 (0.77–1.46)	1.20 (0.86–1.67)	0.27
**Depression**								
Participants/Cases (*n*)	1,071/302	1,071/305	1,071/325		1,071/269	1,072/295	1,072/368	
Crude	1.00	1.01 (0.84–1.22)	1.11 (0.92–1.34)	0.27	1.00	1.13 (0.93–1.37)	1.56 (1.30–1.88)	<0.001
Adjusted^†^	1.00	1.20 (0.95–1.51)	1.27 (1.00–1.61)	0.05	1.00	1.05 (0.83–1.34)	1.22 (0.95–1.57)	0.12
**Distress**								
Participants/Cases (*n*)	1,048/228	1,056/235	1,044/277		1,048/233	1,052/227	1,048/280	
Crude	1.00	1.03 (0.84–1.27)	1.30 (1.06–1.59)	0.01	1.00	0.96 (0.78–1.18)	1.28 (1.04–1.56)	0.02
Adjusted^†^	1.00	1.09 (0.85–1.40)	1.39 (1.09–1.79)	0.01	1.00	0.82 (0.64–1.06)	0.94 (0.72–1.23)	0.68

a *All values are odds ratios and 95% confidence intervals*.

† *Adjusted-model: Adjusted for age, sex, marital status, diabetes, smoking, physical activity, anti-psychotic medications*.

Table 5 reports multivariable-adjusted odds ratios across different categories of ABSI, stratified by sex. After adjusting potential confounders, females in the last tertile of ABSI had an increased odds of anxiety (OR: 1.58; 95%CI; 1.12, 2.22), depression (OR: 1.40; 95%CI; 1.07, 1.84), and psychological distress (OR: 1.51; 95%CI; 1.13, 2.01), compared to the reference tertile. However, there were no significant relations between ABSI and anxiety, depression, and psychological distress among males in the crude and adjusted models. Sex-stratified analysis showed no significant relation between BRI and the outcomes (data not shown).

**Table 5:** Multivariable- adjusted odds ratio for anxiety, depression and distress across tertiles of ABSI, stratified by sex^a^.

	**Men (*****n** **=*** **1,196)**				**Women (*****n** **=*** **2,017)**			
	**Tertiles of ABSI**				**Tertiles of ABSI**			
	**T_**1**_**	**T_**2**_**	**T_**3**_**	**P_**trend**_**	**T_**1**_**	**T_**2**_**	**T_**3**_**	**P_**trend**_**
**Anxiety**								
Participants/Cases (*n*)	269/21	448/37	479/41		802/109	623/112	592/129	
Crude	1.00	1.06 (0.61–1.86)	1.11 (0.64–1.91)	0.72	1.00	1.39 (1.05–1.86)	1.77 (1.34–2.35)	<0.001
Adjusted^†^	1.00	0.90 (0.45–1.80)	0.80 (0.39–1.66)	0.55	1.00	1.35 (0.96–1.90)	1.58 (1.12–2.22)	0.01
**Depression**								
Participants/Cases (*n*)	269/58	448/92	479/96		802/244	623/213	592/229	
Crude	1.00	0.94 (0.65–1.36)	0.91 (0.63–1.32)	0.63	1.00	1.19 (0.95–1.49)	1.44 (1.15–1.80)	0.001
Adjusted^†^	1.00	1.07 (0.66–1.72)	0.97 (0.59–1.57)	0.82	1.00	1.21 (0.92–1.57)	1.40 (1.07–1.84)	0.02
**Distress**
Participants/Cases (*n*)	259/38	437/72	462/88		789/190	619/163	582/189	
Crude	1.00	1.15 (0.75–1.76)	1.37 (0.90–2.07)	0.12	1.00	1.13 (0.89–1.44)	1.52 (1.20–1.92)	0.001
Adjusted^†^	1.00	0.97 (0.58–1.64)	1.07 (0.63–1.82)	0.74	1.00	1.09 (0.82–1.45)	1.51 (1.13–2.01)	0.01

a *All values are odds ratios and 95% confidence intervals*.

† *Adjusted-model: Adjusted for age, marital status, diabetes, smoking, physical activity, anti-psychotic medications*.

Crude and multivariable-adjusted regression coefficients are provided in Table 6. When we considered the exposures (ABSI and BRI) and outcomes (psychological disorders) as the continuous variables, both ABSI and BRI positively predicted anxiety, depression and psychological distress, in the crude model. After adjusting all potential confounders, ABSI was a positive predictor for anxiety (beta coefficient = 0.05, *P* = 0.01), depression (beta coefficient = 0.04, *P* = 0.04) and distress (beta coefficient = 0.05, *P* = 0.02). However, BRI did not significantly predict psychological disorders except for anxiety score (beta coefficient = 0.07, *P* = 0.003).

**Table 6:** Linear association of ABSI and BRI with anxiety, depression and distress scores.

	**ABSI** **× 100**						**BRI**					
	**β^a^**	**B^**b**^**	**P**	**SE^**c**^**	**R-squared**	**VIF^**d**^**	**β^*a*^**	**B**	**P**	**SE**	**R-squared**	**VIF**
**Anxiety score**												
Crude	0.04	0.23	0.01	0.09	0.002	1.00	0.12	0.44	<0.001	0.06	0.02	1.00
Adjusted^†^	0.05	0.29	0.01	0.11	0.09	1.05	0.07	0.24	0.003	0.08	0.09	1.23
**Depression score**												
Crude	0.03	0.12	0.15	0.09	0.001	1.00	0.10	0.33	<0.001	0.06	0.01	1.00
Adjusted^†^	0.04	0.21	0.04	0.10	0.09	1.05	0.03	0.10	0.16	0.07	0.09	1.23
**Distress score**												
Crude	0.04	0.16	0.02	0.07	0.002	1.00	0.07	0.17	<0.001	0.05	0.004	1.00
Adjusted^†^	0.05	0.21	0.02	0.09	0.06	1.05	0.02	0.06	0.33	0.06	0.06	1.23

a *Values are standardized regression coefficients*.

b *Values are unstandardized regression coefficients*.

c *Standard error of the unstandardized regression coefficient*.

d *Variance Inflation Factor that measures the amount of multi-collinearity*.

† *Adjusted-model: Adjusted for age, gender, marital status, diabetes, smoking, physical activity, and anti-psychotic medications*.

## Discussion

In the present cross-sectional investigation among Iranian adults, we observed significant positive associations between ABSI and anxiety, depression, and psychological distress. Such associations were independent of the considered confounders. Sex-stratified analysis revealed significant direct relation between ABSI and the mentioned psychological disorders in females, but not in males. Our study did not support a significant independent relation between BRI and anxiety, depression, and psychological distress after controlling potential confounders. Furthermore, we found that ABSI could positively predict anxiety, depression and distress. BRI could be a positive predictor for anxiety, but not depression and distress. As far as we know, this is the first study exploring the relationship between two novel anthropometric indices (ABSI and BRI) and anxiety, depression, and psychological distress in a large population of Iranian adults.

The global prevalence of psychological disorders and obesity has sharply increased (32). These two conditions have been found to be tightly interrelated (33) and contribute to sleep disorders, the occurrence of chronic diseases, and poor quality of life (11). We observed that ABSI was positively associated with common psychological disorders. Therefore, our findings could provide insights for future randomized controlled trials to address whether reducing ABSI would result in improved mental health. Interventions aiming at either increasing physical activity or reducing calorie intake could potentially decrease ABSI.

We found a direct relation between ABSI and anxiety, depression, and psychological distress. Conversely, we failed to find any significant association between BRI and these outcomes. To the best of our knowledge, only one cross-sectional study has previously examined ABSI in relation to anxiety and depression (34). In the study by Hadi et al. (34), 307 overweight and obese Iranian adults (249 women and 58 men) were enrolled. This study found no significant association between ABSI and anxiety and depression. Several points could explain the discrepancies between our findings and those reported by the mentioned investigation. First, our study sample size was larger, and therefore, the current study has more power to detect the relations. Second, the study by Hadi et al. (34) was conducted among overweight and obese individuals, whereas our investigation included normal- and under-weight subjects, as well. Third, the previous study (34) controlled for the confounding effects of age and sex; however, we considered various other potential confounders in our analysis. Given the lack of evidence regarding the relation between ABSI/BRI and common psychological disorders in other populations, further well-designed studies, especially prospective cohorts, are required among different societies.

Our investigation found a significant positive association between ABSI and anxiety, depression, and psychological distress among females, but not in males. Fewer male participants and, therefore, fewer cases might be a possible reason for the observed insignificant associations. Furthermore, the brain system in women is more sensitive to hormonal fluctuations than in men, which could consequently mediate depressive states (35). Also, women are more vulnerable to depression due to some psychosocial events, including gender-specific socialization, disadvantaged social status, victimization, and internalization coping style (35). We found inverse associations between ABSI and odds of anxiety and depression in men, although the results were not statistically significant. Compared to women, men have more muscle mass, and a previous study has demonstrated a negative association between muscle mass and depressive symptoms (36). However, more researches are needed to elucidate the association between muscle mass and mental health. Furthermore, the “jolly fat” hypothesis might explain the inverse associations in men. In brief, the “jolly fat” hypothesizes that obese men are less likely to have depression or anxiety, possibly due to some nutrients more consumed by obese than non-obese adults (37).

We observed significant positive associations between BRI and anxiety, depression and psychological distress. However, our findings were dependent of age, sex, marital status, history of diabetes, smoking, physical activity levels and use of anti-psychotic medications. Previous studies documented that gender could be a potential determinant for mental disorders; females suffer from depression and anxiety more than males (38, 39). Furthermore, some studies revealed that married individuals were less likely to be depressed (40, 41). Prior studies showed positive relations between having diabetes (42, 43), smoking (44), and low physical activity (45) and risk of anxiety and depression. Therefore, the above-mentioned associations show that various lifestyle and demographic factors might be related to anxiety and depression. This could elucidate the insignificant association between BRI and the outcomes of interest in the adjusted models in the present study.

The positive association between ABSI and common psychological disorders in our study could be clarified through some mechanisms. Obesity and central fatness could induce a low-grade systemic inflammation by increasing plasma levels of inflammatory cytokines (46). Elevated levels of inflammatory cytokines might, in turn, exacerbate depression and anxiety status (47). Furthermore, obesity might lead to hypothalamic-pituitary-adrenal axis (HPA-axis) dysregulation (48, 49). Dysregulation of the HPA-axis has been known to be involved in depression (50). Diabetes and insulin resistance, as consequences of obesity, could involve brain alterations and, in turn, increase mental disorders (51, 52). Along with biological mechanisms, sociocultural factors are also important. In societies that being thin is defined as a beauty ideal and is more socially accepted, overweight and obese individuals have more body dissatisfaction and lower self-esteem, which are risk factors for depression (53).

There are several strengths attributed to the present study. This is the first study that evaluated ABSI and BRI in relation to common psychological disorders including anxiety, depression, and psychological distress in a large sample size of Iranian adults. Validated questionnaires were applied to assess mental health status. Furthermore, we explored the associations in both genders separately. Finally, several potential confounders were taken into account in the analyses to find more independent associations of ABSI and BRI with mental disorders. Despite the above-mentioned strengths, our study has some considerable limitations. First, causal relationships would not be conferred due to the cross-sectional design of the study. Previous literature has strongly suggested a bidirectional relation between obesity and depression (54). Therefore, prospective studies are required to examine the causal relation between ABSI/BRI and common psychological disorders. Second, self-reported anthropometric values have been demonstrated to be less accurate than those obtained by actual measurement (55), although we have previously reported the validity of data provided by self-reported measurements (26). Third, despite considering several covariates in our analyses, some residual confounders might still affect the results. Fourth, we were not able to examine ABSI and BRI against gold-standard tools due to their unavailability. Finally, the population of this study is from the non-academic staff of a medical university, and therefore, findings should be cautiously extrapolated to the general population. As we know, there was no similar study in other nations, therefore, more studies on different countries and populations are warranted.

In conclusion, higher value of ABSI was related to increased odds of anxiety, depression and psychological distress, especially in females. However, there was no association between BRI and anxiety, depression and psychological distress. Also, ABSI was found to be a positive predictor of anxiety, depression and distress. Our results highlighted individuals with visceral adiposity might also suffer from anxiety, depression and distress. Therefore, clinicians should monitor mental health status of overweight/obese patients. This monitoring could lead to prevention or early diagnosis of mental disorders and could subsequently reduce the burden of both conditions. In terms of public health application, ABSI could be a reliable value for developing screening and preventing strategies for abdominal obesity and mental health. Due to the bidirectional relation between adiposity and psychological disorders, prospective cohort studies are worthwhile to affirm the causal relation.

## Data Availability Statement

The original contributions presented in the study are included in the article/Supplementary Materials, further inquiries can be directed to the corresponding author/s.

## Ethics Statement

The studies involving human participants were reviewed and approved by Medical Research Ethics Committee of Isfahan University of Medical Sciences, Isfahan, Iran. The patients/participants provided their written informed consent to participate in this study.

## Author Contributions

KL, AH, PS, HA, AE, and PA contributed to conception, design, statistical analyses, data interpretation, and manuscript drafting. AE supervised the study. All authors approved the final manuscript for submission.

## Funding

This research was supported by Tehran University of Medical Sciences, Tehran, Iran (no. 52283).

## Conflict of Interest

The authors declare that the research was conducted in the absence of any commercial or financial relationships that could be construed as a potential conflict of interest.

## Publisher's Note

All claims expressed in this article are solely those of the authors and do not necessarily represent those of their affiliated organizations, or those of the publisher, the editors and the reviewers. Any product that may be evaluated in this article, or claim that may be made by its manufacturer, is not guaranteed or endorsed by the publisher.

## Abbreviations

ABSI: A body Shape Index
BMI: Body Mass Index
BRI: Body Roundness Index
CVD: Cardiovascular Disease
GHQ: General Health Questionnaire
HADS: Hospital Anxiety and Depression Scale
HPA-axis: Hypothalamic-pituitary-adrenal axis
SEPAHAN: Studying the Epidemiology of Psycho-Alimentary Health and Nutrition
SD: Standard deviation
WC: Waist Circumference
WHR: Waist-hip Ratio.

## References

[1] Vigo D Thornicroft G Atun R. Estimating the true global burden of mental illness. Lancet Psychiatry. (2016) 3:171–8. 10.1016/S2215-0366(15)00505-2 26851330

[2] Mojtabai R. National trends in mental health disability, 1997–2009. Am J Public Health. (2011) 101:2156–63. 10.2105/AJPH.2011.300258 21940913 PMC3222386

[3] Steel Z Marnane C Iranpour C Chey T Jackson JW Patel V. The global prevalence of common mental disorders: a systematic review and meta-analysis 1980–2013. Int J Epidemiol. (2014) 43:476–93. 10.1093/ije/dyu038 24648481 PMC3997379

[4] Depression W. Other Common Mental Disorders: Global Health Estimates. Geneva: World Health Organization (2017).

[5] Santomauro DF Herrera AMM Shadid J Zheng P Ashbaugh C Pigott DM. Global prevalence and burden of depressive and anxiety disorders in 204 countries and territories in 2020 due to the COVID-19 pandemic. Lancet. (2021) 398:1700–12. 10.1016/S0140-6736(21)02143-7 34634250 PMC8500697

[6] Mohamadi M Mohaqeqi Kamal SH Vameghi M Rafiey H Setareh Forouzan A Sajjadi H. meta-analysis of studies related prevalence of depression in Iran. J Res Health. (2017) 7:581–93. 10.18869/acadpub.jrh.7.1.581

[7] Hajebi A Motevalian SA Rahimi-Movaghar A Sharifi V Amin-Esmaeili M Radgoodarzi R. Major anxiety disorders in Iran: prevalence, sociodemographic correlates and service utilization. BMC Psychiatry. (2018) 18:261. 10.1186/s12888-018-1828-2 30126386 PMC6102821

[8] Agarwal A Chauhan K Shinglot K. Anthropometric indices and dietary intake: prospective determinants of geriatric cognitive impairment? Nutr Health. (2013) 22:157–67. 10.1177/0260106014563448 25820204

[9] Ahmadi SM Mohammadi MR Mostafavi S-A Keshavarzi S Kooshesh S-M-A Joulaei H. Dependence of the geriatric depression on nutritional status and anthropometric indices in elderly population. Iran J Psychiatry. (2013) 8:92–6. 24130608 PMC3796300

[10] Ho RC Niti M Kua EH Ng TP. Body mass index, waist circumference, waist–hip ratio and depressive symptoms in Chinese elderly: a population-based study. Int J Geriatr Psychiatry. (2008) 23:401–8. 10.1002/gps.1893 17879255

[11] Pereira-Miranda E Costa PR Queiroz VA Pereira-Santos M Santana ML. Overweight and obesity associated with higher depression prevalence in adults: a systematic review and meta-analysis. J Am Coll Nutr. (2017) 36:223–33. 10.1080/07315724.2016.1261053 28394727

[12] Amiri S Behnezhad S. Obesity and anxiety symptoms: a systematic review and meta-analysis. Neuropsychiatrie. (2019) 33:72–89. 10.1007/s40211-019-0302-9 30778841

[13] Krakauer NY Krakauer JC. A new body shape index predicts mortality hazard independently of body mass index. PLoS ONE. (2012) 7:e39504. 10.1371/journal.pone.0039504 22815707 PMC3399847

[14] Zhao G Ford ES Li C Tsai J Dhingra S Balluz LS. Waist circumference, abdominal obesity, and depression among overweight and obese US adults: National Health and Nutrition Examination Survey 2005-2006. BMC Psychiatry. (2011) 11:1–9. 10.1186/1471-244X-11-130 21834955 PMC3163524

[15] Dong C Sanchez L Price R. Relationship of obesity to depression: a family-based study. Int J Obes. (2004) 28:790–5. 10.1038/sj.ijo.0802626 15024401

[16] Everson-Rose SA Lewis TT Karavolos K Dugan SA Wesley D Powell LH. Depressive symptoms and increased visceral fat in middle-aged women. Psychosom Med. (2009) 71:410–6. 10.1097/PSY.0b013e3181a20c9c 19398501 PMC2739059

[17] Bertoli S Leone A Krakauer NY Bedogni G Vanzulli A Redaelli VI. Association of Body Shape Index (ABSI) with cardio-metabolic risk factors: A cross-sectional study of 6081 Caucasian adults. PLoS ONE. (2017) 12:e0185013. 10.1371/journal.pone.0185013 28945809 PMC5612697

[18] Jung SJ Woo HT Cho S Park K Jeong S Lee YJ. Association between body size, weight change and depression: systematic review and meta-analysis. Br J Psychiatry. (2017) 211:14–21. 10.1192/bjp.bp.116.186726 28428339

[19] Thomas DM Bredlau C Bosy-Westphal A Mueller M Shen W Gallagher D. Relationships between body roundness with body fat and visceral adipose tissue emerging from a new geometrical model. Obesity. (2013) 21:2264–71. 10.1002/oby.20408 23519954 PMC3692604

[20] Maessen MF Eijsvogels TM Verheggen RJ Hopman MT Verbeek AL de Vegt F. Entering a new era of body indices: the feasibility of a body shape index and body roundness index to identify cardiovascular health status. PloS ONE. (2014) 9:e107212. 10.1371/journal.pone.0107212 25229394 PMC4167703

[21] Chang Y Guo X Chen Y Guo L Li Z Yu S. A body shape index and body roundness index: two new body indices to identify diabetes mellitus among rural populations in northeast China. BMC Public Health. (2015) 15:1–8. 10.1186/s12889-015-2150-2 26286520 PMC4544789

[22] Stefanescu A Revilla L Lopez T Sanchez SE Williams MA Gelaye B. Using A Body Shape Index (ABSI) and Body Roundness Index (BRI) to predict risk of metabolic syndrome in Peruvian adults. J Int Med Res. (2020) 48:1–12. 10.1177/0300060519848854 31144540 PMC7140225

[23] Tian T Zhang J Zhu Q Xie W Wang Y Dai Y. Predicting value of five anthropometric measures in metabolic syndrome among Jiangsu Province, China. BMC Public Health. (2020) 20:1–9. 10.1186/s12889-020-09423-9 32867710 PMC7457352

[24] Baveicy K Mostafaei S Darbandi M Hamzeh B Najafi F Pasdar Y. Predicting metabolic syndrome by visceral adiposity index, body roundness index and a body shape index in adults: a cross-sectional study from the Iranian RaNCD cohort data. Diabetes Metab Syndr Obes Targets Ther. (2020) 13:879. 10.2147/DMSO.S238153 32273739 PMC7102908

[25] Adibi P Keshteli AH Esmaillzadeh A Afshar H Roohafza H Bagherian-Sararoudi R. The study on the epidemiology of psychological, alimentary health and nutrition (SEPAHAN): overview of methodology. J Res Med Sci. (2012) 17:S292–8.

[26] Aminianfar A Saneei P Nouri M Shafiei R Hassanzadeh-Keshteli A Esmaillzadeh A. Validity of self-reported height, weight, body mass index, and waist circumference in Iranian adults. Int J Prev Med. (2021) 12:75. 10.4103/ijpvm.IJPVM_422_18 34447517 PMC8356979

[27] Montazeri A Vahdaninia M Ebrahimi M Jarvandi S. The Hospital Anxiety and Depression Scale (HADS): translation and validation study of the Iranian version. Health Qual Life Outcomes. (2003) 1:1–5. 10.1186/1477-7525-1-14 12816545 PMC161819

[28] Brennan C Worrall-Davies A McMillan D Gilbody S House A. The Hospital Anxiety and Depression Scale: a diagnostic meta-analysis of case-finding ability. J Psychosom Res. (2010) 69:371–8. 10.1016/j.jpsychores.2010.04.006 20846538

[29] Montazeri A Harirchi AM Shariati M Garmaroudi G Ebadi M Fateh A. The 12-item General Health Questionnaire (GHQ-12): translation and validation study of the Iranian version. Health Qual Life Outcomes. (2003) 1:1–4. 10.1186/1477-7525-1-66 14614778 PMC280704

[30] Schmitz N Kruse J Heckrath C Alberti L Tress W. Diagnosing mental disorders in primary care: the General Health Questionnaire (GHQ) and the Symptom Check List (SCL-90-R) as screening instruments. Soc Psychiatry Psychiatr Epidemiol. (1999) 34:360–6. 10.1007/s001270050156 10477956

[31] Service NH. The General Practice Physical Activity Questionnaire (GPPAQ)—A Screening Tool to Assess Adult Physical Activity Levels, Within Primary Care. Department of Health (2009).

[32] Pradhan J Dey D Swain M. Relationship between obesity and mental illness. Eur J Mol Clin Med. (2020) 7:1315–22.

[33] Scott KM McGee MA Wells JE Oakley Browne MA. Obesity and mental disorders in the adult general population. J Psychosom Res. (2008) 64:97–105. 10.1016/j.jpsychores.2007.09.006 18158005

[34] Hadi S Momenan M Cheraghpour K Hafizi N Pourjavidi N Malekahmadi M. Abdominal volume index: a predictive measure in relationship between depression/anxiety and obesity. Afr Health Sci. (2020) 20:257–65. 10.4314/ahs.v20i1.31 33402914 PMC7750042

[35] Noble RE. Depression in women. Metabolism. (2005) 54:49–52. 10.1016/j.metabol.2005.01.014 15877314

[36] Gariballa S Alessa A. Associations between low muscle mass, blood-borne nutritional status and mental health in older patients. BMC Nutr. (2020) 6:6. 10.1186/s40795-019-0330-7 32190345 PMC7066831

[37] Palinkas LA Wingard DL Barrett-Connor E. Depressive symptoms in overweight and obese older adults: a test of the “jolly fat” hypothesis. J Psychosom Res. (1996) 40:59–66. 10.1016/0022-3999(95)00542-0 8730645

[38] Faravelli C Scarpato MA Castellini G Sauro CL. Gender differences in depression and anxiety: the role of age. Psychiatry Res. (2013) 210:1301–3. 10.1016/j.psychres.2013.09.027 24135551

[39] Gao W Ping S Liu X. Gender differences in depression, anxiety, and stress among college students: a longitudinal study from China. J Affect Disord. (2020) 263:292–300. 10.1016/j.jad.2019.11.121 31818792

[40] Bulloch AG Williams JV Lavorato DH Patten SB. The depression and marital status relationship is modified by both age and gender. J Affect Disord. (2017) 223:65–8. 10.1016/j.jad.2017.06.007 28732242

[41] Yan XY Huang S Huang C-Q Wu W-H Qin Y. Marital status and risk for late life depression: a meta-analysis of the published literature. J Int Med Res. (2011) 39:1142–54. 10.1177/147323001103900402 21986116

[42] Roy T Lloyd CE. Epidemiology of depression and diabetes: a systematic review. J Affect Disord. (2012) 142:S8–21. 10.1016/S0165-0327(12)70004-6 23062861

[43] Smith KJ Béland M Clyde M Gariépy G Pagé V Badawi G. Association of diabetes with anxiety: a systematic review and meta-analysis. J Psychosom Res. (2013) 74:89–99. 10.1016/j.jpsychores.2012.11.013 23332522

[44] Fluharty M Taylor AE Grabski M Munafò MR. The association of cigarette smoking with depression and anxiety: a systematic review. Nicotine Tobacco Res. (2016) 19:3–13. 10.1093/ntr/ntw140 27199385 PMC5157710

[45] Schuch FB Vancampfort D Firth J Rosenbaum S Ward PB Silva ES. Physical activity and incident depression: a meta-analysis of prospective cohort studies. Am J Psychiatry. (2018) 175:631–48. 10.1176/appi.ajp.2018.17111194 29690792

[46] Park HS Park JY Yu R. Relationship of obesity and visceral adiposity with serum concentrations of CRP, TNF-α and IL-6. Diabetes Res Clin Pract. (2005) 69:29–35. 10.1016/j.diabres.2004.11.007 15955385

[47] Castanon N Lasselin J Capuron L. Neuropsychiatric comorbidity in obesity: role of inflammatory processes. Front Endocrinol. (2014) 5:74. 10.3389/fendo.2014.00074 24860551 PMC4030152

[48] Pasquali R Vicennati V. Activity of the hypothalamic–pituitary–adrenal axis in different obesity phenotypes. Int J Obes. (2000) 24:S47–9. 10.1038/sj.ijo.0801277 10997608

[49] Walker BR. Activation of the hypothalamic-pituitary-adrenal axis in obesity: cause or consequence? Growth Hormone IGF Res. (2001) 11:S91–S5. 10.1016/S1096-6374(01)80015-0 11527096

[50] Belanoff JK Kalehzan M Sund B Fleming Ficek SK Schatzberg AF. Cortisol activity and cognitive changes in psychotic major depression. Am J Psychiatry. (2001) 158:1612–6. 10.1176/appi.ajp.158.10.1612 11578992

[51] Huber J. Diabetes, cognitive function, and the blood-brain barrier. Curr Pharm Des. (2008) 14:1594–600. 10.2174/138161208784705441 18673200

[52] Ajilore O Haroon E Kumaran S Darwin C Binesh N Mintz J. Measurement of brain metabolites in patients with type 2 diabetes and major depression using proton magnetic resonance spectroscopy. Neuropsychopharmacology. (2007) 32:1224–31. 10.1038/sj.npp.1301248 17180124

[53] Luppino FS de Wit LM Bouvy PF Stijnen T Cuijpers P Penninx BW. Overweight, obesity, and depression: a systematic review and meta-analysis of longitudinal studies. Arch Gen Psychiatry. (2010) 67:220–9. 10.1001/archgenpsychiatry.2010.2 20194822

[54] Vittengl JR. Which body shape dimensions and sizes predict depression? J Affect Disord. (2019) 250:193–8. 10.1016/j.jad.2019.03.032 30861461

[55] Gorber SC Tremblay M Moher D Gorber B. A comparison of direct vs. self-report measures for assessing height, weight and body mass index: a systematic review. Obes Rev. (2007) 8:307–26. 10.1111/j.1467-789X.2007.00347.x 17578381

