# Supplementary material for: A Body Shape Index and Body Roundness Index in Relation to Anxiety, Depression, and Psychological Distress in Adults
Source: Front Nutr. 2022 Apr 25;9:843155. doi: 10.3389/fnut.2022.843155 (PMC9083462; doi:10.3389/fnut.2022.843155)
Supplement: Supplementary file 1 [file Table_1.DOCX]

**Supplementary Table 1. Multivariable- adjusted odds ratio for anxiety, depression and distress across tertiles of ABSI and BRI, after excluding anti-psychotic medication users (n=3048) ^1^**

|  | Tertiles of ABSI | | |  | | Tertiles of BRI | | | |  |
| --- | --- | --- | --- | --- | --- | --- | --- | --- | --- | --- |
|  | T_1_ | T_2_ | T_3_ | | P_trend_ | | T_1_ | T_2_ | T_3_ | P_trend_ |
| Anxiety |  |  |  | |  | |  |  |  |  |
| Participants/ Cases (n) | 1026/ 114 | 1018/ 127 | 1004/ 139 | |  | | 1034/ 102 | 1020/ 121 | 994/ 157 |  |
| Crude | 1.00 | 1.16 (0.85-1.59) | 1.32 (0.96-1.81) | | 0.09 | | 1.00 | 1.15 (0.83-1.59) | 1.53 (1.12-2.09) | 0.007 |
| Adjusted^†^ | 1.00 | 1.32 (0.95-1.82) | 1.47 (1.06-2.04) | | 0.02 | | 1.00 | 1.03 (0.74-1.44) | 1.19 (0.84-1.69) | 0.31 |
| Depression |  |  |  | |  | |  |  |  |  |
| Participants/ Cases (n) | 1026/ 274 | 1018/ 275 | 1004/ 286 | |  | | 1034/ 249 | 1020/ 265 | 994/ 321 |  |
| Crude | 1.00 | 1.11 (0.88-1.40) | 1.20 (0.95-1.51) | | 0.13 | | 1.00 | 1.10 (0.87-1.39) | 1.47 (1.17-1.85) | 0.001 |
| Adjusted^†^ | 1.00 | 1.22 (0.96-1.54) | 1.30 (1.02-1.67) | | 0.04 | | 1.00 | 1.03 (0.80-1.31) | 1.17 (0.90-1.52) | 0.23 |
| Distress |  |  |  | |  | |  |  |  |  |
| Participants/ Cases (n) | 1004/ 207 | 1003/ 211 | 977/ 245 | |  | | 1011/ 216 | 1001/ 201 | 972/ 246 |  |
| Crude | 1.00 | 1.01 (0.79-1.30) | 1.34 (1.04-1.71) | | 0.02 | | 1.00 | 0.86 (0.67-1.11) | 1.14 (0.90-1.46) | 0.30 |
| Adjusted^†^ | 1.00 | 1.11 (0.86-1.44) | 1.44 (1.11-1.87) | | 0.01 | | 1.00 | 0.80 (0.61-1.03) | 0.93 (0.71-1.23) | 0.62 |

^1^All values are odds ratios and 95% confidence intervals.

^†^Adjusted-model: Adjusted for age, gender, marital status, diabetes, smoking, physical activity.
